# RepairNatrix: a Snakemake workflow for processing DNA sequencing data for DNA storage

**DOI:** 10.1093/bioadv/vbad117

**Published:** 2023-08-26

**Authors:** Peter Michael Schwarz, Marius Welzel, Dominik Heider, Bernd Freisleben

**Affiliations:** Department of Mathematics and Computer Science, University of Marburg, Marburg 35032, Germany; Department of Mathematics and Computer Science, University of Marburg, Marburg 35032, Germany; Department of Mathematics and Computer Science, University of Marburg, Marburg 35032, Germany; Department of Mathematics and Computer Science, University of Marburg, Marburg 35032, Germany

## Abstract

**Motivation:**

There has been rapid progress in the development of error-correcting and constrained codes for DNA storage systems in recent years. However, improving the steps for processing raw sequencing data for DNA storage has a lot of untapped potential for further progress. In particular, constraints can be used as prior information to improve the processing of DNA sequencing data. Furthermore, a workflow tailored to DNA storage codes enables fair comparisons between different approaches while leading to reproducible results.

**Results:**

We present RepairNatrix, a read-processing workflow for DNA storage. RepairNatrix supports preprocessing of raw sequencing data for DNA storage applications and can be used to flag and heuristically repair constraint-violating sequences to further increase the recoverability of encoded data in the presence of errors. Compared to a preprocessing strategy without repair functionality, RepairNatrix reduced the number of raw reads required for the successful, error-free decoding of the input files by a factor of 25–35 across different datasets.

**Availability and implementation:**

RepairNatrix is available on Github: https://github.com/umr-ds/repairnatrix.

## 1 Introduction

In recent years, using DNA as a storage device has drawn increased attention from academia and industry (Takahashi *et al.* 2019, Press *et al.* 2020, Bee *et al.* 2021, Löchel *et al.* 2021, Schwarz and Freisleben 2021, El-Shaikh *et al.* 2022, El-Shaikh and Seeger 2023, Ezekannagha *et al.* 2023, Pic *et al.* 2023, Welzel *et al.* 2023). DNA is an attractive alternative to traditional storage devices, due to its large storage density of up to $10^{18}\frac{\mathrm{bytes}}{mm^{3}}$, long life expectancy, and low energy requirements for storage (Ceze *et al.* 2019). To store digital data in DNA, the data must be converted into the quaternary DNA alphabet, consisting of the nucleotides Adenine (A), Guanine (G), Cytosine (C), and Thymine (T). Instead of simply assigning each two-bit pair to one of the four bases, it is common to use more sophisticated codes to translate binary data to DNA and add redundancy for error correction. To generate DNA that satisfies desired constraints, such codes often restrict the output codewords to a subset of all possible base combinations of a given length. Common constraints (Schwarz *et al.* 2020) are: (i) Guanine–Cytosine (GC) content of 40%–60%, and no large subsequences that deviate from this range, (ii) limited length of homopolymers (a repetition of the same base), and (iii) no occurrence of specific motifs (short subsequences of a specific pattern), e.g. restriction recognition sites used to cut DNA fragments during synthesis. Furthermore, the codes split the input data into shorter fragments to decrease synthesis costs and ease subsequent sequencing. DNA oligonucleotides (oligos) with the base composition of the generated codewords are generated during synthesis. The chemical synthesis methods often generate only short oligos of 100 nucleotides (nt). If the generated codewords are longer than these limitations, multiple shorter oligos are hybridized using various methods (Kosuri and Church 2014).

Several approaches exist to store the synthesized DNA, e.g. in microwell plates and in lower temperatures to further reduce the probability of depurination (An *et al.* 2014). Alternative storage methods described in the literature are, for example, storing DNA in silicone gel (Grass *et al.* 2015) or integrating the information carrying DNA into living cells, i.e. *in vivo* storage. DNA sequencing methods are used to digitize the DNA again; the Illumina sequencing-by-synthesis approach is commonly used for this purpose. The Illumina approach generates many short reads of up to 150 bp. With paired-end reads, this can increase to 300 bp.

The sequencers generate FASTQ files that contain multiple reads and, for each read, a sequence identifier, the read itself, and a quality score for each base. The quality score, or PHRED score, is encoded as a single ASCII letter. It is related to the probability that a base was called wrongly: *Q* = −10log_10_*P*, where *Q* is the quality score and *P* is the probability that the base was called wrongly by the sequencer. For each sample, either one (single-end) or two (paired-end) FASTQ files are generated. In the paired-end approach, where a single sequence is read from both directions, each forward and reverse read has to be merged to get the full-length read. The resulting FASTQ files consist of different sequences, with multiple reads per unique parent sequence in varying qualities, including sequences with a low quality that are likely wrong. The FASTQ files need extensive processing before the input data can be decoded.

There are several workflows that process raw sequencing data, but no general workflow exists to process sequencing data for DNA data storage. Typically, a custom script (Erlich and Zielinski 2017) or a customized version of a workflow designed for biological data (Welzel *et al.* 2023) is used. We present RepairNatrix, a workflow for processing DNA sequencing data designed for DNA storage. RepairNatrix uses the Snakemake workflow management engine (Köster and Rahmann 2012) to encapsulate each workflow step into self-contained rules with automatic dependency deployment and allows seamless scaling to different computing environments. RepairNatrix contains rules for quality filtering, primer removal, forward- and reverse-read merging, de-replication, optional rules for similarity clustering, assembly of *in vivo* data, and filtering according to sequence constraints. RepairNatrix can repair erroneous sequences using a maximum-likelihood repair algorithm. It leverages knowledge of sequence constraints to infer the most likely originating sequence from an erroneous one. Compared to a preprocessing strategy without repair functionality, RepairNatrix reduced the number of raw reads required for the successful, error-free decoding of the input files by a factor of 25–35 across different datasets. We also show how the sequence design limitations of DNA storage can be used as additional information during the reconstruction of oligonucleotides, thereby improving decodability.

## 2 Approach

DNA sequencing workflows such as QIIME 2 (Estaki *et al.* 2020) or Natrix (Welzel *et al.* 2023) are designed for processing environmental samples, with the goal of clustering and taxonomically assigning corresponding sequencing reads. Since there are various restrictions during *de novo* synthesis, storage, and sequencing for DNA storage, these sequencing workflows are not optimized for this use case.

The status quo for en- and decoding in DNA storage is to treat these restrictions as limitations of the medium, and thus create codes that simply adhere to these rules. Our approach is to use the limitations and restrictions as additional information. From an information theory point of view, the presence or absence of rule violations contains information that can and should be used to retrieve the original data. Using this prior knowledge, we present a DNA processing workflow dedicated to different kinds of DNA data storage, and we provide a code-independent repair approach.

### 2.1 FASTQ files

The primary input of RepairNatrix is (one or multiple) FASTQ files. FASTQ is used as an output format of several sequencing technologies, such as Illumina and Oxford Nanopore sequencing machines. Typically, a FASTQ file consists of multiple entries. Each entry consists of a header line that contains the sequence ID and a description, one line of the sequence itself (the read of the sequencing machine), a line consisting of a plus symbol, and a line containing the PHRED quality information of the read. It is a single ASCII encoded value for each base in a sequence, representing the probability that the base was called wrongly by the sequencer. The PHRED quality score can be calculated from the probability of a wrongly called base ($P_{e}$) as follows (Cock *et al.* 2009): *Q*_PHRED_ = −10 × log_10_($P_{e}$) It is common to generate a single FASTQ file per sample (Sieber *et al.* 2022). This approach can be utilized for DNA data storage by sequencing single or closely related files. Alternatively, several different files can be sequenced in a single sample and subsequently sorted according to a short identifier added to the sequences. This process is known as demultiplexing.

### 2.2 Primer table

Before sequencing, samples are often amplified using polymerase chain reaction (PCR), a fast and cost-efficient approach to duplicate DNA. The PCR process requires the addition of short subsequences (primers) to the amplification target. To remove these primer sequences, together with other additional subsequences that are not part of the encoded data (like barcodes that identify sequences belonging to the same file or poly-N spacers), RepairNatrix uses a primer table that contains the additional subsequences that are present in each FASTQ file, both for the forward reads and (optionally) for the reverse read. The subsequences can also be removed using a length parameter, removing the first *n* bases of each read. An example primer table is shown in the Supplementary Material.

### 2.3 Configuration file

A configuration file is also required. It contains the parameters for each workflow step. Using a single configuration file supports automated processing of several files in parallel, while allowing users to adjust RepairNatrix to their requirements and experimental conditions. An example of a configuration file is provided in the Supplementary Material.

### 2.4 Demultiplexing and initial read filtering

As an optional first step of the workflow, the input can be demultiplexed by sorting all reads according to their barcode specified in the primer table. The raw data are then filtered using PRINSEQ (Schmieder and Edwards, 2011). The filtering process removes all reads with a lower mean PHRED quality score than a user-defined threshold.

### 2.5 Sequence removal, read merging, and de-replication

If additional subsequences from the biochemical processing are still present in the data, they must be removed before further processing. RepairNatrix supports the removal of barcode and primer sequences either by the base composition of the subsequences or by length. The merging of forward- and reverse-reads, which is required for paired-end Illumina data, together with removing additional subsequences, is carried out by PANDAseq (Masella *et al.* 2012). Then, the sequences are de-replicated, i.e. identical sequences are replaced by a single representation containing the abundance information in the header of the read data.

### 2.6 Similarity clustering

RepairNatrix supports optional clustering of sequences by a similarity threshold, using VSEARCH (Rognes *et al.* 2016) and a user-defined similarity threshold. For clustering, the sequences are sorted by frequency, with the most common sequence first, or by quality, with the sequence with the highest mean PHRED quality score first. In each iteration of the clustering, the first sequence is removed from the pool of remaining sequences and used as a new cluster’s representative sequence. Each sequence in the pool with a higher similarity than the user-defined threshold (with a default value of 97%) to the representative of the current cluster is added to this cluster and removed from the pool of available sequences. This approach is commonly used to generate operational taxonomic units in microbiome analysis (Welzel *et al.* 2020). Here, it serves to merge sequences that differ slightly, using the sequence with either the highest quality (i.e. the sequence with the highest confidence of the sequencer) or the most common sequence as the most likely correct sequence. The similarity threshold should be adjusted to reflect the minimal Hamming distance of two encoded sequences with the used error-correcting code.

### 2.7 Filtering constraint-violating sequences

RepairNatrix can (optionally) remove sequences that violate user-defined constraints, which is carried out after merging forward- and reverse-reads. Since many DNA storage codes can not only add redundancy to the input for error correction purposes but can also generate outputs that adhere to specific constraints, reads that contain subsequences that could not be generated by the codes can, optionally, be filtered out from further processing. Removing these reads can lead to an increase in error correction performance, especially if, for example, early during PCR amplification an error occurred that led to the formation of an erroneous subsequence, like a long homopolymer. This could lead to the wrong sequence being designated as the representative sequence of the cluster. With the erroneous reads filtered out, less potential errors to be corrected by the error correcting code remain, increasing the probability of successful decoding. Since repairing the sequences requires prior similarity clustering, filtering allows users to remove erroneous sequences, even if users do not want the data to be clustered or repaired. This feature will ensure that all processed sequences adhere to the restrictions defined by a user, but it is not possible to use the repair features afterwards. Filtering is divided into two main modes. In the first mode, it can operate as an input filter, removing constraint-violating sequences. This approach ensures that the downstream decoding receives only valid sequences according to the coding restrictions. While this approach does not introduce any additional incorrect sequences and thus cannot reduce the dataset’s quality, some coding schemes might be able to reconstruct the correct data from sequences that violate the restrictions. For these codes, removing these sequences could lower the number of unique sequences in the dataset and thus prevent successful decoding. To accommodate this scenario, RepairNatrix can be used in a second mode to sort the sequences regarding compliance with the rules. In this mode, a weight is applied to each rule violation, together with the (mean) quality score for each (clustered) sequence. Thus, decoding algorithms for coding schemes with unordered encoded data (such as fountain codes) will successfully reconstruct the encoded file before reading low-quality and rule-violating sequences with a higher probability.

### 2.8 Maximum-likelihood repair

One of the key features of RepairNatrix is its ability to perform a maximum-likelihood repair of constraint-violating sequences. The repair functionality requires user-defined constraints that the encoded sequences must adhere to. Such constraints are: (i) global and per-window GC-content, (ii) maximum allowed homopolymer length, (iii) k-mer length, (iv) undesired subsequences, and (v) length of the sequence.

RepairNatrix comes with pre-defined rules, but its Snakemake-based design allows users to add additional rules. The pre-defined rules can be adjusted by modifying the .yaml file of the project. In contrast, custom rules require modifying the calc_errors function to include user-created functions in the detection and repair process. Such user-defined rules could include support for functions like checksums, unique constraints, or secondary structure predictions. This flexibility allows RepairNatrix to improve the recovery rate for all constraint-satisfying coding schemes.

The maximum-likelihood repair algorithm of RepairNatrix replaces the base with the highest likelihood of an error by the base, which would minimize the overall error prediction of the current sequence. Depending on the number of rules and mutations, this can be a heuristic approach, but it is very unlikely that the final output will be worse than without maximum-likelihood repair. Users can opt to keep the constraint-violating sequence in the set of all sequences. At the same time, the modified sequence will also be flagged with additional information about the number of changes performed to this sequence.

**Figure 1.**
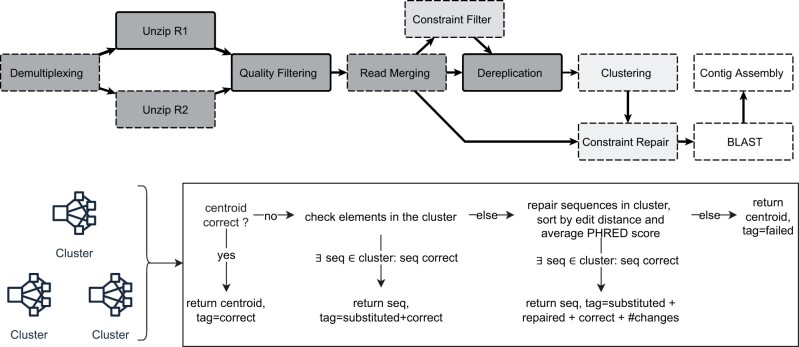
Top: Overview of the RepairNatrix main sequence processing modules. Nodes with dashed borders are optional. Dark nodes represent raw sequence processing steps, light grey nodes are related to the constraint-based output improvements, and white nodes are *in vivo* specific. Bottom: Workflow for sequence selection/repair for each cluster.

As shown in Fig. 1, the repair algorithm performs a series of operations on each cluster. First, each centroid of a cluster will be checked against the defined rules. If the sequence adheres to the rules, the centroid will be treated as correct and tagged accordingly. Otherwise, each element in the cluster will be analyzed regarding the user-defined rules (sorted by their edit distance to the centroid). If such a sequence exists, it replaces the current centroid and will be tagged accordingly. If no such sequence exists in the current cluster, the algorithm tries to perform maximum-likelihood repair on the sequences in the cluster (sorted by their edit distances to the centroid). For this purpose, the algorithm applies all user-defined rules to create an error metric for each base in the sequence, then it modifies the base with the highest error metric to minimize the overall error of the sequence, as well as the error metric of the chosen base. This process might continue for up to *n* bases, where *n* is a user-defined threshold after which a sequence will be treated as not recoverable. By default, this algorithm will perform such a repair for all sequences in a cluster, filter out all sequences for which the repair failed, and return the sequence with the lowest number of changes introduced during the repair.

**Figure 2.**
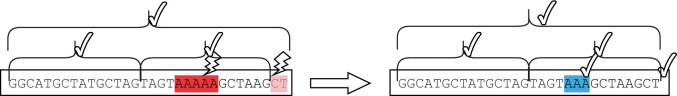
A sequence with a homopolymer of length 5, no GC content violations, and a sequence length of 32. The algorithm correctly flags all positions with a homopolymer and detects that the length of the sequence is longer than allowed. The corrected sequence has neither a homopolymer longer than 3, and the sequence length is 30.

**Figure 3.**
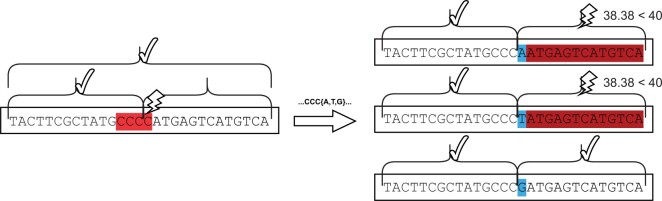
A sequence with an invalid homopolymer running between two GC windows. The maximum likelihood repair iterates over the homopolymer from the last to the first position to find a suitable repair path. While substituting the last *C* with *A* or *T* would violate the windowed GC content constraint, a substitution with *G* yields a valid solution.

Fig. 2 and 3 show a simplified example of possible repairable sequences. For these sequences, we assume an overall and windowed (per 15 bases) GC content of [40, 60], a maximum allowed homopolymer length of 3, as well as a fixed sequence length of 30.

### 2.9 *In vivo* filtering and assembly

To process data stored *in vivo*, RepairNatrix offers filtering by user-provided genomic data. For *in vivo* data storage, the raw reads typically contain a mixture of payload data and genomic data from the host, or data from the used vector. If the payload data are not extracted from the mixture of reads, the decoding could fail, even in the presence of a sufficiently large number of raw payload reads. With a provided database consisting of the genome of the host organism, RepairNatrix uses the basic local alignment tool (BLAST) to separate the input data into two files: one containing reads that are likely part of the host genome and do not contain stored data, and a second one containing the reads that either did not match any target of the database or only match with low confidence (i.e. a high *E*-value), which are likely sequences belonging to the encoded data. To support shotgun-style sequencing, in which the genome is cloned and randomly fragmented, RepairNatrix uses SPAdes (Prjibelski *et al.* 2020) to assemble fragments to larger contigs, combined with knowledge regarding the length of the encoded data sequences, or if short spacer sequences are used to identify the start and end of encoded data. This approach supports the reconstruction of the encoded sequences from sequencing data consisting of a mixture of the encoded data fragments and other sequences (e.g. host genomic data or artificial chromosomes).

## 3 Evaluation

We evaluated RepairNatrix’s ability to improve sequence processing output for DNA storage using the data and evaluation metrics published by (Welzel *et al.* 2023).

**Figure 4.**
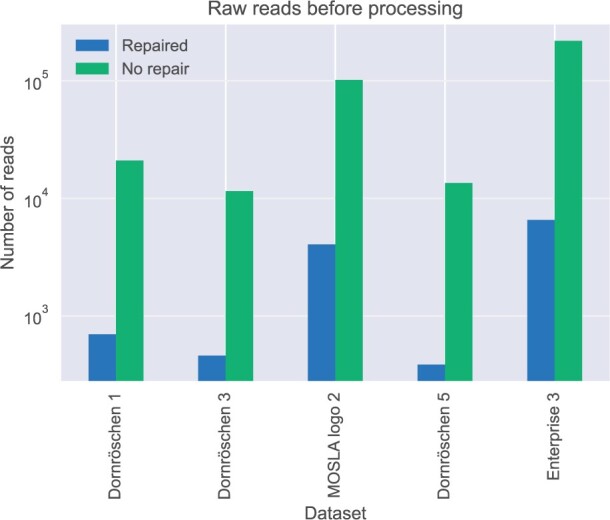
The minimal number of raw reads required for successful decoding before processing. The numbers after the dataset names describe the CRC interval parameter used for the encoding.

The complete results are shown in the Supplementary Material. The evaluation was carried out for a mean quality threshold of 10 and a PANDAseq quality score of 0.3. This combination led to the lowest required number of sequences in the results published previously (Welzel *et al.* 2023). Using RepairNatrix, the number of raw reads needed for the successful, error-free decoding of the input files was between 25 (Dornröschen, CRC interval 2 and 3) and 35 (Dornröschen, CRC interval 5) times lower using the repair and cluster representative choosing algorithms of RepairNatrix than without, as shown in Fig. 4.

**Figure 5.**
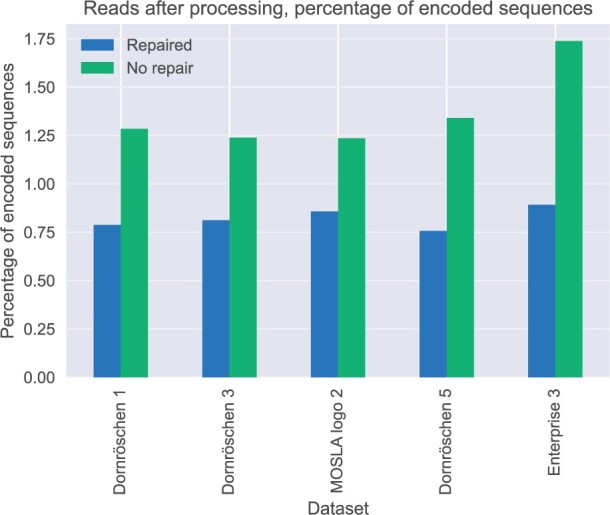
The minimal amount of sequences left for successful decoding after processing. The numbers after the dataset names describe the CRC interval parameter used for the encoding.

The lowest number of sequences after processing that were required for successful decoding were 77%–89% of the encoded sequences before synthesis, as shown in Fig. 5. This implies that most of the sequences left after processing were descendants of unique encoded sequences and not just erroneous variants of the same encoded sequence. The results shown in Figs 4 and 5 demonstrate that incorporating prior constraints into the preprocessing leads to successful decoding with either fewer raw reads or reads that contain a higher percentage of erroneous sequences.

**Figure 6.**
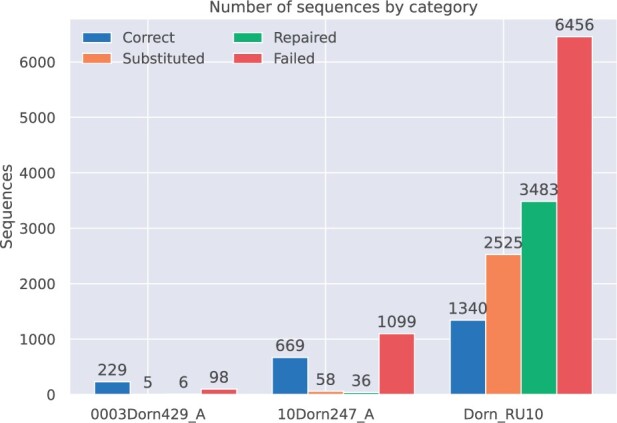
The distribution of each sequence category for different experiments. For low overhead (01), only a fraction of all sequences violate the constraints. For four clusters, a rule abiding substitute could be found.

To analyze the behavior of the proposed method, we compared the resulting sequence categories. For the *in vitro* experiments using 247 and 429 chunks, the coverage was reduced to the corresponding percentage (e.g. 0.003%, 10% of the raw sequenced reads). For the Dorn_RU10 in-silico experiment, a more restrictive ruleset was enforced during creation of the 184 nt long sequences. This included limiting the maximum allowed homopolymer count to 3, blacklisting 57 sequences from 6 nt to up to 35 nt, as well as enforcing a global and windowed GC content between 40% and 60%. Figure 6 shows the number of sequences for each output category per experiment. For the more restrictive *in silico* experiment, for which we also introduced a higher number of indel errors and substitution errors using the MESA simulator (Schwarz *et al.* 2020), the number of repaired or substituted sequences significantly increases. Information about the repaired sequences, the affected cluster, and their difference will be stored in a json file for each processed file.

**Figure 7.**
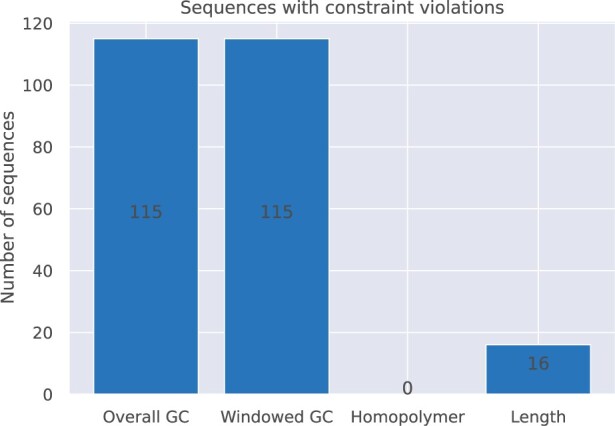
Different constraint violations for the *in vitro* experiment (Sleeping Beauty) reduced to 10% of its original coverage.

Figure 7 shows the found and corrected rule violations for the parsed sequences. Besides the shown rules, no violation toward undesired sequences was found. While this is mainly due to the small list of undesired subsequences (5 sequences between 19 and 35 nt each), the absence of homopolymer errors can be explained by the (mostly) error-free synthesis, short storage duration, and robust sequencing technology used. Therefore, most errors detected were based on a constraint-violating (windowed) GC content as well as a non-matching sequence length.

To further evaluate the effectiveness of RepairNatrix, we compared using it for raw sequence file preprocessing with the preprocessing method described in the DNA-Fountain publication, where the authors were able to successfully decode data using a random selection of 750 000 raw Illumina sequencing reads. We downloaded the raw data and, after confirming that using both RepairNatrix and the preprocessing approach described by the authors, the data could be successfully decoded using a random selection of 750 000 reads, we reduced the input data to 700 000 randomly selected sequencing reads and preprocessed the reads with both strategies, followed by decoding the preprocessed data using DNA-Fountain. Using the approach described in the DNA-Fountain publication, 123 326 sequences of length 152 nucleotides were left after preprocessing. The decoding reported success, but the MD5 hash of the encoded file did not match the MD5 hash of the input data, indicating a failure to decode the data correctly. An inspection of the output file indicated that it was corrupted. With RepairNatrix, 125 283 sequences of length 152 nucleotides were left after preprocessing. The decoding resulted in a file with the MD5 hash of the input data, as described in the Supplementary Material of the DNA-Fountain publication. The decoded file was a gzip compressed tarball containing multiple files, including an image of the KolibriOS, a PDF of Shannon’s Mathematical Theory of Communication (Shannon 1948), and a 3 pg video.

## 4 Conclusion

RepairNatrix is a Snakemake-based read-processing workflow that combines Natrix’s raw sequencing data processing capabilities with maximum likelihood reconstruction algorithms tailored to DNA data storage, the ability to extract payload data from a mixture of genomic sequences and payload sequences, and filtering of data with user-defined constraints. This combination allows easy and fast recovery of data stored in DNA, agnostic to the coding scheme used for translating binary data to DNA. The low-code configuration and setup of RepairNatrix allow users to easily restore the most likely sequences for DNA storage experiments from a pool of raw sequencing data. Besides in-vitro storage, RepairNatrix also supports *in vivo* storage, allowing users to blast against a database consisting of host-genome data to filter out biological sequences from encoded sequences and to assemble shotgun sequencing data into contigs. In addition, RepairNatrix processes Illumina and Oxford Nanopore data. Since RepairNatrix is written in the workflow management engine Snakemake, with all dependencies encapsulated in rule-specific Conda (https://docs.conda.io/en/latest/) environments, results can easily be reproduced, allowing a comparison of different coding methods with the same preprocessing, including the same version for every tool used. All configuration entries are written in a single configuration file, making it easy to share and adjust. In its current version, RepairNatrix can be used with almost any code that requires some form of preprocessing of raw reads before decoding. Codes directly utilizing any form of majority voting during the decoding would most likely only benefit from the basic quality control, read assembly, dereplication and, possibly, from the filtering of constraint-violating sequences. An example of such a code is described in Tabatabaei Yazdi *et al.* (2015). However, such codes could integrate the maximum-likelihood repair into their workflow to profit from our approach. Additionally, the proposed method could be adapted for any code using non-canonical restrictions by implementing and adding these restrictions into the ruleset. For codes that generate sequences with a dependency between each other (e.g. a guaranteed minimal Hamming distance), a more sophisticated maximum-likelihood approach would have to be implemented taking all possible clusters into account. This would be possible using the RepairNatrix architecture, but it is currently not implemented.

## Supplementary Material

vbad117_Supplementary_Data

## Data Availability

The source code of RepairNatrix is available at https://github.com/umr-ds/RepairNatrix. The data underlying this article are available as supplementary data of the mentioned articles (Welzel *et al.* 2023 and Erlich and Zielinski 2017).
