# Supplementary material for: RepairNatrix: a Snakemake workflow for processing DNA sequencing data for DNA storage
Source: Bioinform Adv. 2023 Aug 26;3(1):vbad117. doi: 10.1093/bioadv/vbad117 (PMC10941317; doi:10.1093/bioadv/vbad117)
Supplement: vbad117_Supplementary_Data [file vbad117_supplementary_data.pdf]

Bioinformatics
doi.10.1093/bioinformatics/xxxxxx
Advance Access Publication Date: Day Month Year
Manuscript Category

Subject Section

Supplemental Data for RepairNatrix - a Snakemake workflow for processing DNA sequencing data for DNA storage

Peter Michael Schwarz [1,*], Marius Welzel [1], Dominik Heider, and Bernd Freisleben [*]

Department of Mathematics and Computer Science, University of Marburg, Marburg, 35032, Germany

[*] To whom correspondence should be addressed.
[1] These authors contributed equally to this work.

Associate Editor: XXXXXXX

Received on XXXXX; revised on XXXXX; accepted on XXXXX

Abstract

Motivation: There has been rapid progress in the development of error-correcting and constrained codes for DNA storage systems in recent years. However, improving the steps for processing raw sequencing data for DNA storage has a lot of untapped potential for further progress. In particular, constraints can be used as prior information to improve the processing of DNA sequencing data. Furthermore, a workflow tailored to DNA storage codes enables fair comparisons between different approaches while leading to reproducable results.
Results: We present RepairNatrix, a read-processing workflow for DNA storage. RepairNatrix supports preprocessing of raw sequencing data for DNA storage applications and can be used to flag and heuristically repair constraint-violating sequences to further increase the recoverability of encoded data in the presence of errors. Compared to a preprocessing strategy without repair functionality, RepairNatrix reduced the number of raw reads required for the successful, error-free decoding of the input files by a factor of 25 to 35 across different datasets.
Availability: RepairNatrix is available on Github: https://github.com/umr-ds/repairnatrix
Contact: schwarzk@uni-marburg.de , freisleben@uni-marburg.de

© The Author 2022. Published by Oxford University Press. All rights reserved. For permissions, please e-mail: journals.permissions@oup.com

1 Primer table

RepairNatrix requires a primer table that is structured as shown in Table 1

Probe	poly_N	Barcode_forward	specific_forward_primer	poly_N_rev	Barcode_reverse	specific_reverse_primer
Dorn247_A			TCCATTGCGTCAACCGTTAT			TGCCAATGGAAGTTTCGTGA
Dorn276_A			TACCGCATCCTTATTCGAGC			TTTCATTGGCTTGCACCAGA
Dorn429_A			TCCTGCTTGCGTTAAATGGA			TGCCAATAAGTCTTGCGGAA
MOSLA_A			AATGTCGAAGAAAGCCGGTT			ACCGTGCTTCAAACCGAATT

Table 1. Example primer table, containing the file names (without file extensions and read identifier, i.e., R1, R2), poly_N spacer, Barcodes and primers used for each sample.

2 Example configuration file with default values

For the configuration of sub-workflows and applications used by RepairNatrix, a single configuration file is used. Optional parts of the workflow (e.g., the generation of quality reports, clustering of OTUs, or the assignment of taxonomic information) can be disabled. The configuration file is also used to define the protocols used to generate the input data, e.g., whether the data is in single-end or paired-end format and whether a split-sample approach was used during sample preparation. It also contains configuration options to adjust individual parts of the workflow depending on the requirements of the project. An example configuration file with a description and default value for each parameter is shown in Table 2.

Option	Default	Description
filename	project	The filename of the project folder, primertable (.csv) and config file (.yaml).
primertable	project.csv	Path to the primertable.
units	units.tsv	Path to the sequencing unit sheet.
cores	4	Amount of cores available for the workflow.
demultiplexing	False	Demultiplexing for reads that were not demultiplexed by the sequencing company (slow).
read_sorting	False	Read sorting for paired end reads that were not sorted by the sequencing company (slow).
already_assembled	False	Skipping of the quality control and read assembly steps for data that is already assembled.
in-vivo	False	If the data is unprocessed in-vivo data, that still contains host genonomic data.
threshold	0.9	PANDAseq score threshold a sequence must meet to be kept in the output.
minoverlap	15	Sets the minimum overlap between forward and reverse reads.
minqual	1	Minimal quality score for bases in an assembled read to be accepted by PANDAseq.
minlen	100	The minimal length of a sequence after primer removal to be accepted by PANDAseq.
maxlen	600	The maximal length of a sequence after primer removal to be accepted by PANDAseq.
primer_offset	False	Using PANDAseq to remove primer sequences by length offset instead of sequence identity.
mq	25	Minimum quality sequence check (prinseq), filtering of sequences according to the PHRED quality score before the assembly.
barcode_removed	True	Boolean that indicates if the sequence is free of barcodes.
all_primer	True	Boolean that indicates if the sequence is free of any kind of additional subsequences (primer, barcodes etc.).
clustering_id	1.0	Percent identity for cdhit (dereplication) (1 = 100%), if cdhit is solely to be used for dereplication (recommended), keep the default value.
clustering	True	If clustering should be used.
centroid_selection	frequency	If the sequences should be ordered by frequency or by quality for the clustering.
minsize	2	Minimal size of a cluster to be kept in the output.
length_overlap	0.0	Length difference cutoff, default 0.0 if set to 0.9, the shorter sequences need to be at least 90% length of the representative of the cluster.
paired_End	True	The format of the sequencing data, TRUE if the reads are in paired-end format.
name_ext	R1	The identifier for the forward read (for the reverse read the 1 is switched with 2, if the data is in paired-end format), has to be included at the end of the file name, before the file format identifier (including for single end files).
swarm	True	Boolean to indicate the use of the SWARM clustering algorithm to create operational taxonomic units (OTUs) from the data.
blast_bd	True	Key, Value pairs of the sample postfix and which database path should be used for the in-vivo filtering.

clustering	True	If cdhit dereplication should be used.
inplace_repair	False	Replace sequences that do not adhere to constraints instead of adding a repaired copy
maximum_repair_cycles	100	Threshold of permutations to perform before a sequence will be treated as corrupt
repair_quality_score	33	PHRED score of repaired bases (used for fastq output)
repair_after_demultiplexing	False	Repairing of sequences that do not adhere to constraints after demultiplexing.
repair_after_quality_control	False	Repairing of sequences that do not adhere to constraints after quality control.
repair_after_assembly	False	Repairing of sequences that do not adhere to constraints after assembly.
use_quality_mapping	True	use quality values from fastq reads for repair of (demultiplexed) reads instead of the repair_quality_score
after_demultiplexing	False	Removal of sequences that do not adhere to constraints after demultiplexing.
after_quality_control	False	Removal of sequences that do not adhere to constraints after quality control.
after_assembly	False	Removal of sequences that do not adhere to constraints after read assembly (forward- and reverse-read merging).
primer_length	20	Length of the used primer.
homopolymer_count	7	Length of the longest homopolymer that can occur in the sequences.
windowed_gc_content	gc_min: 0.4, gc_max: 0.6, window_size: 50	The minimal and maximal gc content that can appear in a specific window size.
undesired_subsequences	path	path to a list of motifs or subsequences that should not occur in the sequences.
kmer_counting	k:10, upper_bound: 20, active: false	Length and highest allowed number of occurrences of kmers.

Input file	R1 raw	R2 raw	Encoded sequences	Sequences after processing	Processed multiple of encoded	Raw sequence numbers	Raw multiple of encoded	Percentage of raw reads used
Dornröschen, CRC interval 1	2,327,536	2,327,536	429	551	1.284	20,948	48.82983682983683	0.9
Dornröschen, CRC interval 3	1,151,987	1,151,987	276	342	1.239	11,520	41.73913043478261	1
MOSLA logo, CRC interval 2	2,027,755	2,027,755	1,951	2,409	1.235	101,388	51.96719630958483	5
Dornröschen, CRC interval 5	1,930,388	1,930,388	247	331	1.34	13,513	54.7085020242915	0.7
Enterprise, CRC interval 3	2,180,802	2,180,802	2,704	4,700	1.738	218,080	80.6508875739645	10

Table 2. Results using the files, tools, and parameters as described in (Welzel et al., 2023), for a mean quality threshold of 10 and a PANDAseq quality threshold of 0.3. R1/R2 raw represents the total amount of sequences in the unprocessed FASTQ files. Encoded sequences represent the number of sequences generated by the DNA-Aeon encoder, which were used for the synthesis. Raw multiple of encoded is the number of raw reads as a multiple of the number of encoded sequences required for successful decoding. Processed multiple of encoded represents the minimal amount of assembled reads after processing required for successful decoding. Raw sequence numbers represent the total number of sequences before processing needed for decoding, and percentage of raw reads used is the percentage of reads from the initial FASTQ file used for processing.

Input file	R1 raw	R2 raw	Encoded sequences	Sequences after processing	Processed multiple of encoded	Raw sequence numbers	Raw multiple of encoded	percentage of raw reads used
Dornröschen, CRC interval 1	2,327,536	2,327,536	429	338	0.788	698	1.627039627039627	0.03
Dornröschen, CRC interval 3	1,151,987	1,151,987	276	224	0.812	461	1.6702898550724639	0.04
MOSLA logo, CRC interval 2	2,027,755	2,027,755	1,951	1,672	0.857	4056	2.078933880061507	0.2
Dornröschen, CRC interval 5	1,930,388	1,930,388	247	187	0.757	386	1.562753036437247	0.02
Enterprise, CRC interval 3	2,180,802	2,180,802	2,704	2,411	0.892	6542	2.419378698224852	0.3

Table 3. Results using RepairNatrix, for a mean quality threshold of 10 and a PANDAseq quality threshold of 0.3. R1/R2 raw represents the total amount of sequences in the unprocessed FASTQ files. Encoded sequences represent the number of sequences generated by the DNA-Aeon encoder, which were used for the synthesis. Raw multiple of encoded is the number of raw reads as a multiple of the number of encoded sequences required for successful decoding. Processed multiple of encoded represents the minimal amount of assembled reads after processing required for successful decoding. Raw sequence numbers represent the total number of sequences before processing needed for decoding, and percentage of raw reads used is the percentage of reads from the initial FASTQ file used for processing.

References

Welzel, M. *et al.* (2023). DNA-Aeon provides flexible arithmetic coding for constraint adherence and error correction in DNA storage. *Nature Communications*, **14**(1).
